# Deep Plane Facelift “Bucket Handle” Technique Video

**DOI:** 10.1093/asjof/ojaf079

**Published:** 2025-06-26

**Authors:** Manoj T Abraham, Megan Tang, Solomon Husain

## Abstract

The deep plane facelift isolates the superficial musculoaponeurotic system (SMAS) by direct lysis of retaining ligaments, allowing greater mobilization of the vascularized composite flap of skin and SMAS. The authors of this study describe a modification to the deep plane facelift, termed the “bucket handle” technique. This technique involves creating a triangle of SMAS tissue extending from the angle of the mandible posteriorly to the anterior aspect of the sternocleidomastoid muscle. The intact SMAS flap, which is not divided or myotomized, is draped over the angle of the mandible, augmenting the gonial angle, whereas the triangular bucket handle of the SMAS flap is sutured back to the mastoid crevasse, enhancing the posterior medial vector of the neck lift, providing a more sculpted, defined jawline.

**Level of Evidence: 5 (Therapeutic)**: 
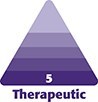

Facelift techniques have evolved from superficial musculoaponeurotic system (SMAS) plication pioneered by Skoog, to the high SMAS approach originally described by Barton, ultimately leading to the composite deep plane lift by Hamra, with extended versions of the deep plane lift developed by Warren and subsequently popularized by Jacono.^1-5^ Traditionally, the deep plane entry point is created along a diagonal line between the angle of the mandible and the lateral canthus of the eye, marking the transition zone between the mobile anterior face and the fixed lateral face (Figure 1).^3,6,7^ Alternative deep plane entry points have been proposed to address limitations of the standard technique. Sand et al describe entering the deep plane at the high SMAS facelift incision line, which begins superior to the zygomatic arch and angles posteriorly at the anterior ear, advancing past the platysma muscle of the neck. This incision produces a more dramatic bidirectional lift of the SMAS and skin.^2,8^ Other studies extend the deep plane entry line from the angle of the mandible into the neck for joint SMAS–platysma suspension, resulting in significant neck rejuvenation.^5,9^

**Figure 1.**
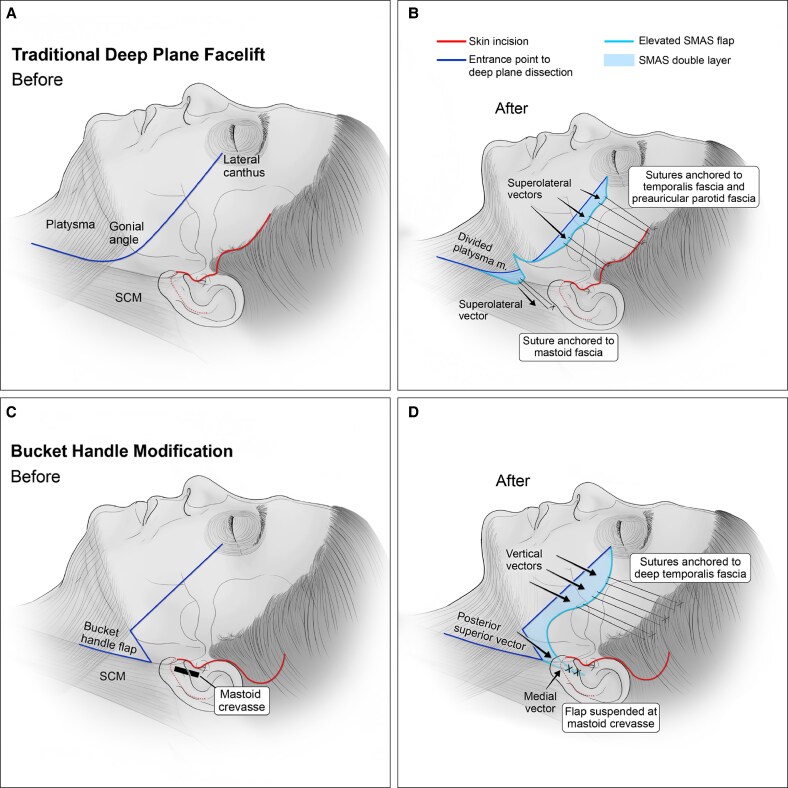
Illustration depicting the traditional deep plane facelift technique (A, B) and our bucket handle modification (C, D). SCM, sternocleidomastoid muscle.

Our “bucket handle” technique is a novel and effective approach within extended deep plane facelift practices (Video). The key step involves forming a bucket handle of cervical SMAS extending from the angle of the mandible posteriorly to the sternocleidomastoid muscle (SCM) and inferiorly down the SCM, which is then suspended posteriorly and medially at the mastoid crevasse to tighten the neck and define the jawline (Figure 2). The bucket handle obviates the need to divide the SMAS flap which can be layered over the angle of the mandible to provide gonial angle augmentation and achieve a more natural contour.

**Figure 2.**
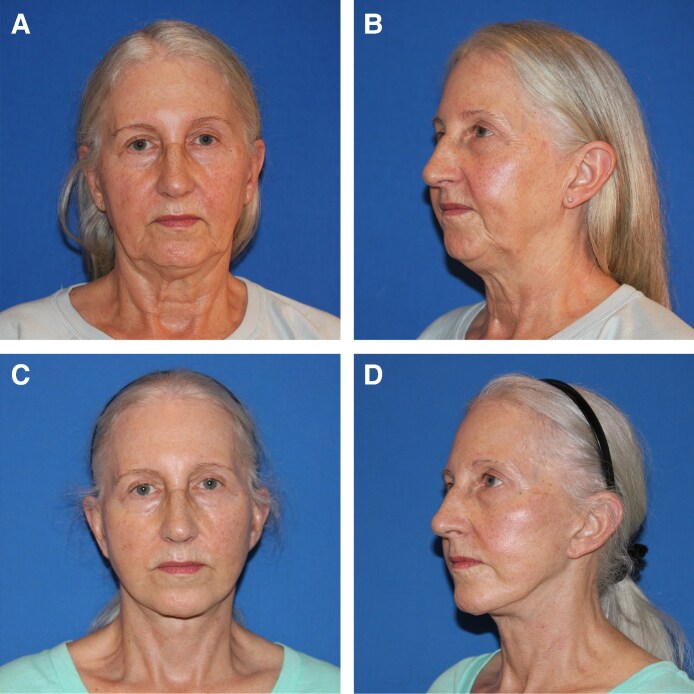
Representative 72-year-old female patient seeking facial rejuvenation before (A, B) and 1 year after (C, D) facelift using our bucket handle modification.

We employed our bucket handle technique regardless of the medial approach to the neck. When required, submental neck liposuction, digastric muscle and submandibular gland resection, and medial platysma plication were performed before the lateral approach. These medial interventions did not impede utilization of our bucket handle technique and only served to enhance improvements in the neck. Variation in mandible bone anatomy at the gonial angle and mastoid crevasse also did not substantially alter the implementation of this technique, although the exact 3-dimensional anatomy (length and width of the flap, amount of medialization possible) is determined by individual patient anatomy.

This technique does require more dissection and understanding of the anatomy. The senior author has employed this technique in 130 patients over the past 5 years without any significant complications and with uniform success, with patients noting improved definition at the angle of the jaw compared with before the implementation of this technique (Figure 2).

## SURGICAL TECHNIQUE

### Subcutaneous Flap Elevation

The incision begins in the temporal hairline and proceeds downward past the root of the helix to form the preauricular border. The incision then loops around the bottom lobe of the ear and proceeds superiorly in the postauricular crease. The subcutaneous flap is then elevated along this incision 1 cm past the deep plane entry point.

Along with skin elevation, the surgeon dissects bluntly along the anterior border of the mastoid above the mastoid tip and safely away from the facial nerve trunk to expose the mastoid crevasse, the soft tissue space between the anterior border of the mastoid and the posterior border of the mandible below the external auditory canal.^10^ Anchoring our bucket handle within the mastoid crevasse will provide the posterior medial lift of the neck to further define the jaw.

### Deep Plane Elevation

The deep plane entry line is the diagonal path from 1 cm below the lateral canthus of the eye to 1 cm anterior to the angle of the mandible, then extending posteriorly to the SCM before extending inferiorly 4 to 5 cm along the anterior border of the SCM. This creates an angled SMAS flap posteriorly along the jawline, which constitutes the bucket handle. The bucket handle is raised sharply by releasing the deep cervical retaining ligaments off the SCM. Care is taken to remain anterior to the greater auricular nerve. Branches of the external jugular vein may occasionally need to be ligated. The formation of this bucket handle in cervical dissection is the primary difference between our technique and other extended deep plane facelifts.^2-10^

The deep plane is elevated up to the facial artery, starting first with blunt dissection along the easily separable glide plane above the jawline. A subplatysmal plane of dissection is achieved, and the deep cervical fascia is released using blunt dissection to protect nerve branches. Superiorly below the orbicularis, the zygomatic cutaneous ligaments are released sharply, and dissection proceeds above the zygomatic muscles to protect the nerve supply. The masseteric ligaments are then released completely over the parotid and masseteric fascia.

### Flap Suspension

The bucket handle provides a more natural contour by allowing bidirectional suspension of the face and neck without the need to divide the SMAS flap. The superior aspect of the SMAS flap is layered over the zygoma and suspended superiorly by anchoring to the deep temporalis fascia, which minimizes displacement of the ear.^11^ The bucket handle flap at the angle of the mandible is then pulled posteriorly and suspended medially to the deep periosteum within the mastoid crevasse using buried interrupted 3.0 Polydioxanone suture (PDS II; Ethicon, J&J MedTech). The SMAS flap is then layered over the unelevated native SMAS over the angle of the mandible to create a double layer and closed in a tension-free manner with running 4.0 Polyglactin suture (Vicryl Rapide; Ethicon, J&J MedTech). The skin is tailored and closed in a tension-free manner.

## Supplementary Material

ojaf079_Supplementary_Data
